# The Black Box effect: sensory stimulation after learning interferes with the retention of long-term object location memory in rats

**DOI:** 10.1101/lm.053256.120

**Published:** 2021-10

**Authors:** Daisy Arkell, Isabelle Groves, Emma R. Wood, Oliver Hardt

**Affiliations:** 1 Centre for Discovery Brain Science, School of Medicine, The University of Edinburgh, Edingurgh, Scotland EH8 9XD, United Kingdom; 2 The Simons Initiative for the Developing Brain, The Patrick Wild Centre, The University of Edinburgh, Edingurgh, Scotland EH8 9XD, United Kingdom; 3 Department of Psychology, McGill University, Montréal, Quebec H3A 1G1, Canada

## Abstract

Reducing sensory experiences during the period that immediately follows learning improves long-term memory retention in healthy humans, and even preserves memory in patients with amnesia. To date, it is entirely unclear why this is the case, and identifying the neurobiological mechanisms underpinning this effect requires suitable animal models, which are currently lacking. Here, we describe a straightforward experimental procedure in rats that future studies can use to directly address this issue. Using this method, we replicated the central findings on quiet wakefulness obtained in humans: We show that rats that spent 1 h alone in a familiar dark and quiet chamber (the Black Box) after exploring two objects in an open field expressed long-term memory for the object locations 6 h later, while rats that instead directly went back into their home cage with their cage mates did not. We discovered that both visual stimulation and being together with conspecifics contributed to the memory loss in the home cage, as exposing rats either to light or to a cage mate in the Black Box was sufficient to disrupt memory for object locations. Our results suggest that in both rats and humans, everyday sensory experiences that normally follow learning in natural settings can interfere with processes that promote long-term memory retention, thereby causing forgetting in form of retroactive interference. The processes involved in this effect are not sleep-dependent because we prevented sleep in periods of reduced sensory experience. Our findings, which also have implications for research practices, describe a potentially useful method to study the neurobiological mechanisms that might explain why normal sensory processing after learning impairs memory both in healthy humans and in patients suffering from amnesia.

One of the most puzzling phenomena of memory is that we forget, and since its beginning as a scientific discipline, psychology has been trying to find out why and how this happens (Ribot 1882; Ebbinghaus 1885; Müller and Pilzecker 1900; Burnham 1903)? Addressing this question, Jenkins and Dallenbach (1924) published a remarkable study in 1924 suggesting that much forgetting arises from continued mental activity caused by ongoing everyday experiencing that normally follows learning in natural settings. Their intriguing findings were not systematically pursued during the next decades, as the focus shifted to exploring the role of prior or subsequent learning on forgetting; that is, effects of proactive or retroactive interference of highly similar material on memory retention. This research program eventually led into a dead end (Tulving and Madigan 1970; Wixted 2004), and interference research in humans slowed down in the 1970s. In recent years, however, interest about the neurobiological bases of interference began to emerge again (Appleby and Wiskott 2009; Bartko et al. 2010; Blake et al. 2010; Butterly et al. 2012; Luu et al. 2012; Martínez et al. 2012; Winocur et al. 2012; Peters et al. 2013; Alber et al. 2014; Censor et al. 2014; Martínez et al. 2014; McDevitt et al. 2014; Albasser et al. 2015; Eugenia et al. 2016; Koen and Rugg 2016; Ge et al. 2019; Peters and Smith 2020).

In their original experiment, Jenkins and Dallenbach (1924) used sleep to reduce the amount of interference after learning. They found that when their participants went about their normal (university campus) day after learning a list of nonsense syllables, their ability to recall the lists 1, 2, 4, or 8 h later was always poorer than when instead they slept during the time between learning and test. Jenkins and Dallenbach (1924) concluded that their results “indicate that forgetting is not so much a matter of the decay of old impressions and associations than a matter of the interference, inhibition, or obliteration of the old by the new.” Their findings were replicated by others, confirming that being asleep, compared with being awake and active, indeed improves memory retention (Van Ormer 1932; Ekstrand 1967). However, it remained an open question whether it is the reduction of sensory stimulation and new learning, which would usually occur during wakefulness, that prevents retroactive interference, or whether a specific, possibly sleep-dependent, memory facilitation process was at play (Ekstrand 1967, 1972).

Noting that participants in the sleep condition did not immediately fall asleep in the original experiment, but that they experienced increased quiescence shortly after learning, Minami and Dallenbach (1946) tested the retroactive interference explanation of forgetting more directly, by controlling the amount of stimulation after learning in awake animals. This remarkable experiment used *Periplaneta americana* (American cockroach) and a little treadmill. After learning to suppress their natural tendency to run into a dark shelter box in a bright open alley (encouraged by an electrical shock received in the dark shelter), the cockroaches were either placed on a running treadmill in a transparent box, or in a normally lit circular transparent resting chamber, where they were not able to fall asleep but experienced notably less activity than the cockroaches on the treadmill. The outcome was that cockroaches who were forced to move presented with more forgetting than those who were not, suggesting that sleep—notwithstanding its possible beneficial effect on memory—may not be necessary to promote memory retention; rather, reducing the amount of stimulation and activity after learning may be critical for attenuating retroactive interference and thus forgetting.

Some six decades later, a series of experiments picked up this original line of inquiry. Exploring in humans whether memory for short prose, word lists, or spatial knowledge benefits from reduced stimulation after learning, these studies have invariably replicated the main finding that spending a 10-min retention interval in quiet wakefulness in a dimly lit room after learning leads to better memory for the learned material than participating in unrelated cognitive tasks during the retention interval (Dewar et al. 2007, 2010). Increased memory for the acquired material following quiet wakefulness is long-lasting and can be detected up to 7 d after learning (Dewar et al. 2012; Alber et al. 2014). Even in amnesic patients 10 min of reduced sensory stimulation, compared with participating in cognitive tasks, enhances memory retention for verbal material (Cowan et al. 2004; Dewar et al. 2009, 2010). This lends strong support to the suggestion that the memory loss in amnesia arises from an increased vulnerability to interference shortly after encoding (Warrington and Weiskrantz 1974; Hardt et al. 2013)

Similar results have been obtained in rodents in studies exploring the role of perirhinal cortex in object recognition memory. Rats with lesions to the perirhinal cortex typically show robust impairments in object recognition tasks (Brown and Aggleton 2001; Mumby et al. 2002, 2007; Norman and Eacott 2005; Albasser et al. 2015). However, if rats are placed into a dark box during the retention interval between the encoding phase and the test phase of an object recognition task, rats with lesions to perirhinal cortex no longer show a memory deficit and perform as well as intact animals (McTighe et al. 2010). Thus, reduction of sensory stimulation between encoding and test appears to enhance memory for objects even in rats with perirhinal cortex lesions. This finding recapitulates the outcomes of the studies with human patients suffering from amnesia after hippocampal damage.

The aim of the current experiments was to determine whether reducing sensory stimulation after encoding would also enhance hippocampus-dependent memory in rats. To do this, we used a spontaneous object exploration task that assesses memory for object locations (Ennaceur and Delacour 1988; Hardt et al. 2010; Migues et al. 2016, 2019). Using this approach, we replicated in rats the basic effect that quiet wakefulness promotes memory retention as previously observed in humans. Specifically, here we show that following learning, everyday activity in the home cage with cage mates impairs object location memory in rats, while reducing sensory stimulation in a dark chamber, without sleep, promotes it.

## Results

### Sensory stimulation after learning impairs the retention of memory for object locations

**Figure 1.**
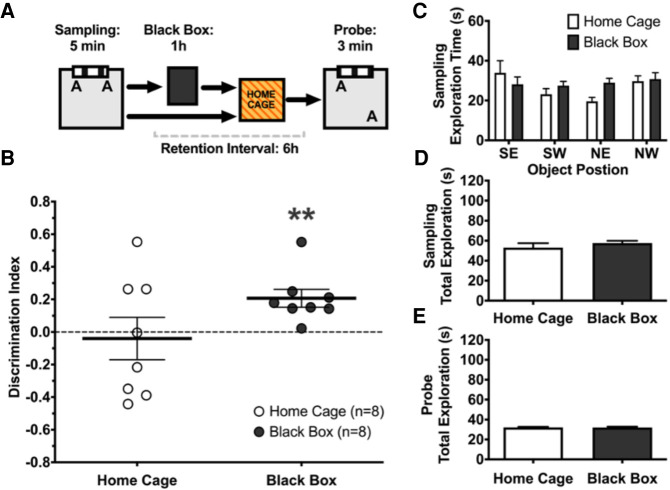
Reducing sensory stimulation after learning promotes retention of long-term object location memory. (*A*) Behavioral protocol of the novel object location recognition task. After the 5-min Sampling phase, we placed rats either into the Black Box or back into their home cage. We tested long-term memory for object locations 6 h after Sampling in the 3-min-long Probe phase. (*B*) Average discrimination index (*d*) during the Probe phase. Rats in the Black Box condition expressed long-term memory for object locations 6 h after Sampling (*d* = 0.207, SD = 0.154), while rats in the Home Cage condition had forgotten them (*d* = −0.0401, SD = 0.368). Each circle represents the discrimination index of an individual animal. (*C*) Time spent exploring objects in different locations during Sampling. Rats had no preference for a location in either condition. (*D*,*E*) Total time exploring objects during Sampling (*D*), and during Probe (*E*). There were no differences between the conditions in either phase. Error bars indicate ±1 SEM. (**) *P* < 0.01.

To test whether reducing sensory stimulation promotes memory retention in rats, we used a version of the spontaneous object exploration task that assesses long-term memory for object locations (Ennaceur and Delacour 1988). We exposed rats during the Sampling phase for 5 min to a familiar open field in which two copies of a novel object were placed in two adjacent corners (Fig. 1B). Then rats returned either to their cage mates in their home cage or were placed into a highly familiar dark, quiet box (The Black Box) for 1 h, and then returned to their home cage. We tested long-term memory for object locations 6 h after learning, returning the rats to the open field in the Probe phase, where we had moved one of the former objects to a novel location (one of the other corners). Rats are naturally attracted to novelty, and thus their preference to explore the moved object rather than the one at the familiar place reflects memory for the original object locations. Based on the findings with humans on the effects of quiet wakefulness on memory retention, we predicted that rats exposed to The Black Box after learning would retain memory for object locations better than rats that returned immediately to their home cage.

Novelty preference in the Black Box condition was not normally distributed (Shapiro–Wilk test, *W* = 0.81, *P* = 0.03) so we analyzed it with a Wilcoxon rank test. As predicted, animals in the Black Box condition (*n* = 8) significantly preferred exploring the object at the novel location during the probe trial (*W* = 36, *P* = 0.008, *r*_*b*_ = 1.0), whereas animals in the home cage condition (*n* = 8) did not, and explored both object locations a similar amount (*t* < 1) (Fig. 1C). The two groups spent a similar total amount of time exploring objects during sampling (*t* < 1) and the probe trial (*t* < 1). Therefore, group differences in motility, motivation or learning cannot readily account for differences in novelty preference. The variances between the two groups were significantly different (*F*_(7,7)_ = 5.68, *P* = 0.04), so we used an unpaired *t*-test with Welch's correction to compare novelty preference between the two groups. This revealed that the groups were not significantly different from each other (*t*_(9.391)_ = 1.749, *P* = 0.11, *d* = 0.88, η_p_^2^ = 0.246), but suggested a statistical trend.

Our post-hoc power analysis revealed that our test was slightly underpowered (1 − β error probability of 0.37), suggesting that a minimum of twelve observations per group would be required to detect significant effects. Furthermore, the absence of a significant group difference on the background of memory retention in the rats exposed to the Black Box might indicate that the Sampling phase was too short to produce a sufficiently robust memory representation to allow for a better separation of the two groups. Therefore, for the following experiments, we increased the number of rats and doubled the Sampling time, assuming this would yield a stronger effect. To decrease the variance between groups we also used a within-subjects design, testing all rats in both conditions in a pseudorandom order. In addition, we tested whether visual stimulation impaired memory retention in rats returned to the home cage, as this form of interference was greatly reduced in the Black Box.

### Visual stimulation after learning impairs the retention of memory for object locations

**Figure 2.**
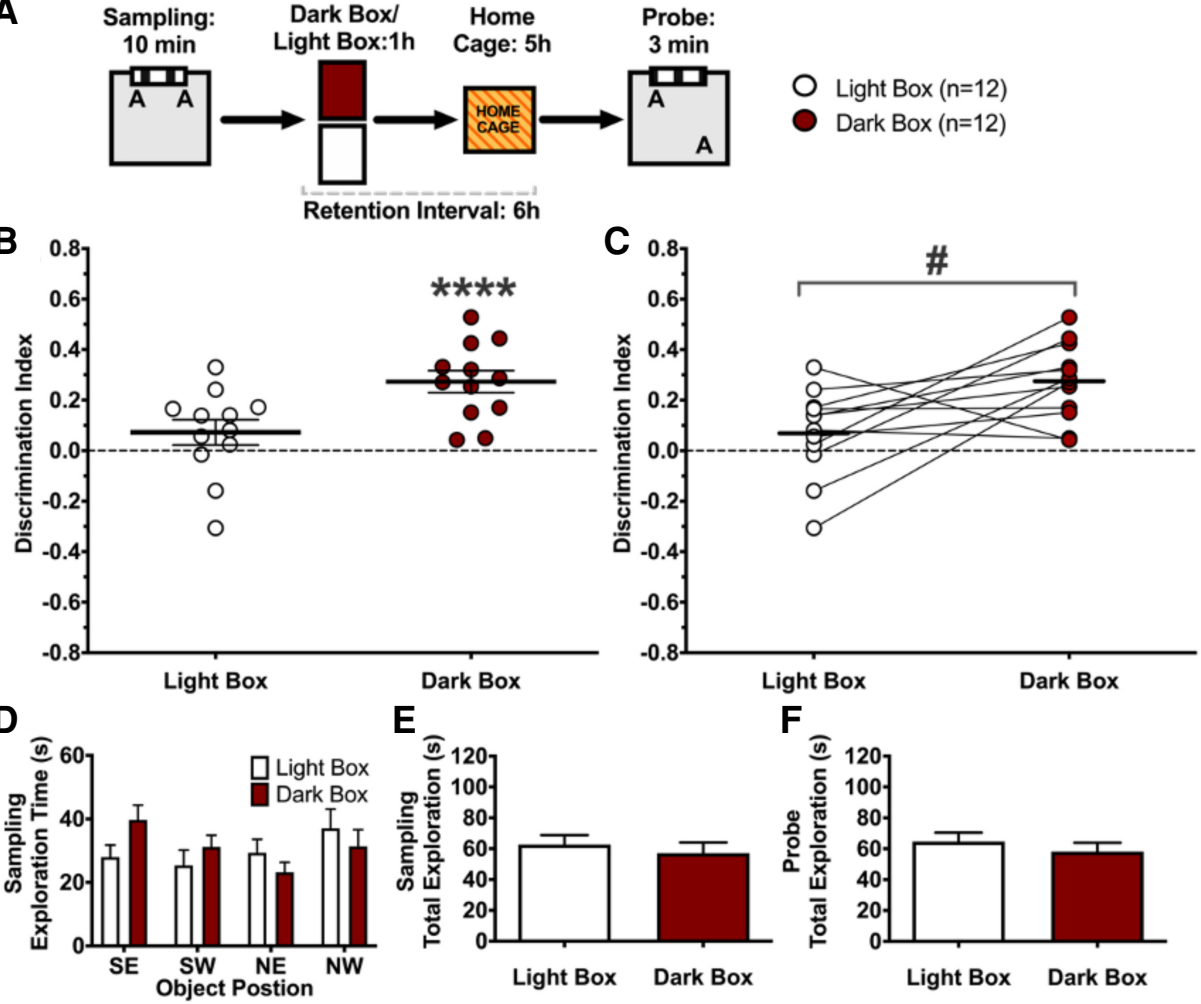
Visual stimulation after learning impairs the retention of memory for object locations. (*A*) Behavioral protocol. We placed rats into a box that was either dark (red light) or normally lit (incandescent light) after the Sampling phase of the novel object location recognition task. Each rat participated twice in this task, once in each condition, with a delay of 2 wk between experiments. (*B*) Average discrimination index (*d*) during the Probe trial. When placed into the Dark Box, rats expressed long-term location memory, exploring the object moved to a new location more than the object at the familiar location (*d* = 0.273, SD = 0.152), but when they were placed into the Light Box they explored both locations to the same extent (*d* = 0.0724, SD = 0.173), suggesting they forgot the object locations. (*C*) Same as *B*, identifying the performance of individual rats in both conditions. There was a significant difference in the preference to explore the object at the novel location between the two conditions. (*D*) Time spent exploring objects in different locations during sampling. No location preference was shown for either condition. (*E*,*F*) Total time spent exploring objects was the same in both conditions during Sampling (*E*) and Probe (*F*). Error bars indicate ±1 SEM. (****) *P* < 0.0001, (#) *P* < 0.05.

The home cage provides a variety of stimuli of various modalities that could potentially interfere retroactively with the new memory for object locations. We first tested whether the Black Box prevented interference because it attenuated visual stimulation (Fig. 2A). First, rats (*n* = 12) participated in the Sampling phase, as in experiment 1, but with a longer sampling period of 10 min. Immediately thereafter, we placed rats for 1 h into a familiar plastic cage housed in a familiar sound attenuating box, which was either illuminated with white light or red light. Rats cannot perceive red light and therefore did not receive visual stimulation in the red light (Dark Box) condition, whereas in the Light Box condition, which had normal illumination, they did receive visual stimulation. A camera in the box allowed us to observe the rats and to prevent sleep with gentle handling. Rats returned to their home cage immediately after the 1-h period in the box, and we tested object location memory 5 h later; that is, 6 h after Sampling. Each rat was tested four times, twice in the Light Box condition and twice in the Dark Box condition (see the Materials and Methods).

Replicating our main result from experiment 1, when rats spent 1 h after Sampling in effective darkness (Dark Box; i.e., red light illumination), they significantly preferred exploring the object moved to a novel location than the object that remained at the familiar place (*t*_(11)_ = 6.24, *P* < 0.0001, *d* = 1.8, η_p_^2^ = 0.779) (Fig. 2B). In contrast, when rats were in the same box under normal illumination (Light Box), they did not show such a preference and explored both objects to a similar extent (*t*_(11)_ = 1.45, *P* = 0.18, *d* = 0.42, η_p_^2^ = 0.16). The difference in novelty preference between the Dark Box and Light Box conditions was significant (paired *t*-test: *t*_(11)_ = 2.70, *P* = 0.021, *d* = 0.78, η_p_^2^ = 0.399) (Fig. 2B). Difference in motility or motivation cannot account for the enhanced novelty preference in the Dark Box because total object exploration was the same in both conditions during both Sampling (*t*_(11)_ = 1.00, *P* = 0.34) (Fig. 2E), and Probe (*t*_(11)_ = 1.02, *P* = 0.33) (Fig. 2F). Furthermore, during Sampling the rats did not show a preference for exploring one of the two object locations in either condition (main effect of location *F*_(3,86)_ = 1.56, *P* = 0.205, interaction between location and condition *F*_(3,86)_ = 1.91, *P* = 0.133) (Fig. 2D), so location preferences during Sampling are unlikely to explain a preference for the novel location during the Probe. Finally, we analyzed whether the order in which rats were exposed to the Dark Box and Light Box modulated performance. A repeated measures ANOVA with box (Dark Box vs. Light Box) and repetition (first vs. second) as repeated factors and sequence of conditions ([Light Box, Dark Box]–[Dark Box, Light Box] vs. [Dark Box, Light Box]–[Light Box, Dark Box]) as between-subjects factor detected a significant effect of box (*F*_(1,10)_ = 6.9, *P* = 0.03, η_p_^2^ = 0.41), with no other main effect or interaction reaching significance (all *F* < 1). Therefore, the sequence in which rats experienced the Dark Box and Light Box cannot explain why being in the Dark Box after Sampling promoted memory retention.

In summary, these findings show that light stimulation after learning is sufficient to disrupt long-term memory for object locations in rats.

### Exposure to a cage mate after learning impairs retention for memory of object locations despite reduced visual stimulation

**Figure 3.**
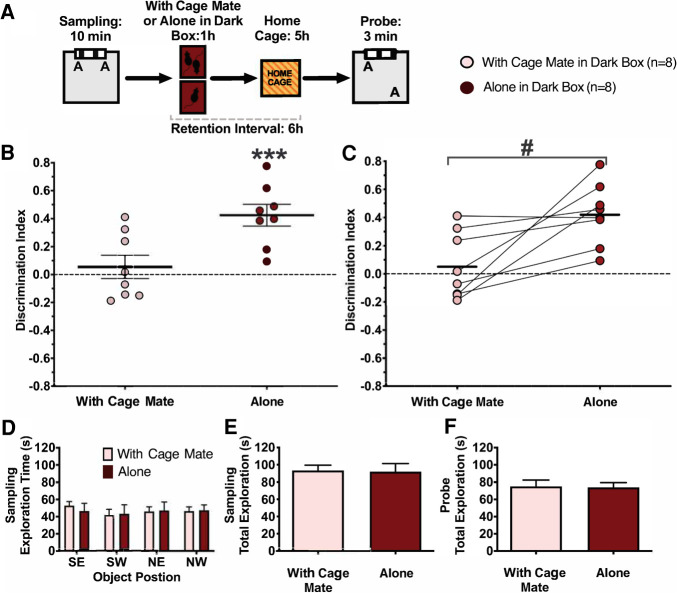
Being with a cage mate in the Dark Box after learning impairs the retention of memory for object locations. (*A*) Behavioral protocol. Immediately after Sampling in the novel object location recognition task, we placed rats into the Dark Box (red light) either alone or with a familiar cage mate. We tested memory for the location of the objects 6 h after Sampling. Rats participated in both conditions, with a delay of 2 wk between experiments. (*B*) Average discrimination index (*d*) during the Probe phase. When rats were alone in the dark box they expressed long-term memory for the object locations, exploring the object moved to a new location more than the object at the familiar location (*d* = 0.425, SD = 0.220); when they were in the dark box together with a cage mate, they did not (*d* = 0.0549, SD = 0.236). (*C*) Same as *B*, identifying the performance of individual rats in both conditions. There was a significant difference in preference exploring the object at the novel location between the two conditions. (*D*) Time spent exploring objects in different locations during sampling. No location preference was shown for either condition. (*E*,*F*) Total time spent exploring objects was the same in both conditions during Sampling (*E*) and Probe (*F*). Error bars indicate ±1 SEM. (***) *P* < 0.001, (#) *P* < 0.05.

A second type of stimulation that the Black Box eliminated, and that might have resulted in retroactive interference, was linked to interacting with other rats in the home cage after learning. In order to test this possibility, we repeated experiment 2 with a further eight rats, with the only difference that rats spent the 1 h after Sampling in the Dark Box either alone or together with a cage mate (within-subjects design) (Fig. 3A). Both conditions reduce visual stimulation to the same extent as in experiment 2, so any differences in memory retention can be attributed to the presence or absence of a conspecific. In this experiment, rats received two trials—one in each condition.

Replicating the main outcome of experiment 2, rats significantly preferred to explore the object moved to a novel location during the Probe trial when they spent the 1 h after Sampling alone in the dark (*t*_(7)_ = 5.46, *P* = 0.0009, *d* = 1.93, η_p_^2^ = 0.81). However, this was not the case when they were with a cage mate during this time, as under this condition the rats explored both objects a similar amount (*t* < 1) (Fig. 3B). The difference between these two conditions was significant (paired *t*-test, *t*_(7)_ = 3.21, *P* = 0.015, *d* = 1.14, η_p_^2^ = 0.596) (Fig. 3B). As in the previous experiments, there were no differences between conditions in total object exploration during Sampling or Probe (*t* < 1 for both) (Fig. 3E,F), ruling out the possibility that differences in motivation, motility, or learning account for the preserved location memory in the rats that remained alone in the Dark Box. As in the previous experiments, rats did not show a preference for either object location during Sampling in either condition (all effects *F* < 1) (Fig. 3D), so location preferences are unlikely to explain the differences between the conditions observed during the Probe. We analyzed possible order effects using a repeated-measures ANOVA with condition (alone vs. cage mate) as the repeated factor and sequence (alone first vs. social first) as the between-subject factor. The ANOVA revealed a significant main effect of condition (*F*_(1,6)_ = 10.1, *P* = 0.02, η_p_^2^ = 0.63), but detected no significant effect of sequence or a significant interaction (for both *F* < 1). Therefore, preserved location memory in the animals that were alone compared with being with a cage mate in the Dark box cannot be explained by the order in which rats experienced these two conditions.

Taken together, these results demonstrate that interacting with another rat after learning is sufficient to disrupt long-term memory for object locations in rats.

## Discussion

We aimed to replicate in rats the well-established finding obtained in humans that reducing sensory stimulation and thus attenuating mental activity after learning promotes long-term memory retention. We found that putting rats alone into a highly familiar dark box in which sensory stimulation was reduced for 1 h after they had explored two copies of an object in an open field resulted in long-term retention of the locations of these objects. In contrast, rats that went directly into the home cage with cage-mates after exploring the objects forgot where the objects had been placed before (experiments 1 and 2). Our findings also show that being together with another cage mate in the dark leads to memory loss (experiment 3). Our results support the conclusion that in rats, like in humans, various types of sensory stimulation after learning can impair long-term memory for the learned material.

These findings extend results from reducing sensory stimulation in rats with lesions to perirhinal cortex (McTighe et al. 2010) in three important ways. First, they show that sensory deprivation enhances memory in a hippocampus-dependent object location task, indicating that this effect is not specific to perirhinal cortex-dependent object recognition memory. Second, they indicate that not only visual stimulation, but also interaction with a conspecific is sufficient to disrupt long-term memory for object locations. Third, they show that sensory deprivation enhances memory in control (nonlesioned) rats, consistent with the earlier findings in control (nonamnesic) human subjects.

What occurs during wakeful resting that promotes memory, which everyday experiences in the period after learning can easily disrupt? Over the years, two main answers to this question have been proposed: memory consolidation and retroactive interference. The former explanation, developed by Ribot (1882) and later in a slightly different form by Müller and Pilzecker (1900), argues that after encoding, transient metabolic or mnemonic processes, respectively, are required to stabilize the newly acquired memory to retain it in the long term. Any disruption of these processes can lead to memory loss. A rich set of findings suggests that these processes include morphological changes to synaptic connections (Lamprecht and LeDoux 2004), likely brought about by perseverating reactivation of the new memory trace in form of hippocampal replay (Ylinen et al. 1995; Buzsáki 1996; Ego-Stengel and Wilson 2010), and possibly requiring nuclear or local synthesis and degradation of proteins (Abel and Lattal 2001; Alberini 2009), their post-translational modifications (Sans et al. 2003; Derkach et al. 2007), as well as epigenetic changes (Sultan and Day 2011). Based on this view, the detrimental effect of ongoing sensory stimulation after learning on memory retention has been explained as reflecting limits in the capacity of concurrent processing in the hippocampus (Wixted 2004), while others have argued that it may result from disrupted or reduced hippocampal replay (Craig et al. 2016a; Craig and Dewar 2018). Both accounts can explain why wakeful rest promotes memory retention. On the other hand, as Jenkins and Dallenbach (1924) suggested, ongoing sensory stimulation may somehow overwrite newly formed memories, thereby causing retroactive interference. At least implicitly, however, this account would need to assume that a process akin to memory consolidation is also involved, because after some time has passed new memories are immune to this form of postlearning erasure. In other words, these two positions might not be mutually exclusive.

Our results seem to be in line with the basic idea that reducing incoming sensory stimulation after learning promotes undisturbed (hippocampal) processing, possibly replay, thus increasing the probability that a newly encoded memory will be retained in the long term. Furthermore, our findings lend support to earlier results in humans that it does not matter so much what type of sensory stimulation is encountered after learning—visual stimulation or interacting with another rat in the dark both impair the retention of newly learned spatial knowledge in our animals. The original report of Jenkins and Dallenbach (1924) similarly suggests that random, everyday experiences arising from a variety of sensory stimuli consistently lead to memory impairment compared with the absence of such events during sleep. Likewise, recent research shows that, compared with quiescent waking, the sensory similarity or type of mental activity elicited by activities during the time between verbal learning and recall does not moderate the amount of forgetting in humans: There was no difference in correct recall of words between participants asked to attentively listen to talk radio, watch a video, perform a visual search task, solve math problems as fast as possible, or detect piano notes masked by brown noise (Dewar et al. 2007).

That the retention of newly learned verbal material can be affected by material of any sensory modality processed shortly thereafter may suggest that a common memory hub, such as the hippocampus, which receives input from areas processing any type of sensory modality, may be involved, lending support to the positions discussed above. Our study, however, did not directly test the role of particular sensory modalities in disrupting memory for object locations. While we eliminated visual stimulation, it remains to be addressed whether introducing other specific forms of sensory stimulation (e.g., an auditory or olfactory stimulus) during the time in the dark also leads to memory loss. The fact that being together with another rat in the Dark Box promoted forgetting of location memories could suggest that various forms of sensory stimulation can cause memory interference, including olfactory, tactile, and auditory processing. Our data, however, cannot determine whether these stimuli lead to forgetting because they affect consolidation by, for example, reducing available hippocampal resources, possibly limiting replay, or whether they are a source of distraction, stress, or emotional arousal that impair memory formation.

Our findings extend those obtained in rats with lesions to perirhinal cortex, in which reducing sensory stimulation after exploring objects prevented amnesia for these objects (McTighe et al. 2010). This effect has been interpreted within the hierarchical-representational model of object memory (Murray and Bussey 1999; Bussey et al. 2005; Bartko et al. 2007a; Saksida and Bussey 2010; Forwood et al. 2012; Kent et al. 2016). Briefly, this theory assumes that objects are represented across all levels of the visual processing stream, spanning early visual processing areas and perirhinal cortex. This projection pathway is thought to form an ordered stack of layers of neural networks, with earlier layers representing more concrete features of visual objects (e.g., lines with orientations), which are integrated into more abstract conjunctions of features in successive layers (e.g., several lines with orientations are integrated into a corner representation). At the end of this processing stream, the perirhinal cortex represents integrations of conjunctions of conjunctions, that is, a highly abstract code for an object that, in its totally, is represented across all layers of this organized network, thereby “binding” the distributed representations into a coherent whole.

This model assumes that representational components for objects overlap more in earlier than in later network layers, and that in perirhinal cortex such overlap will be absent as it, in a sense, represents the unique identity of objects in a highly abstract binding code. Damage to the perirhinal cortex will lead to deficits in object recognition because the natural interference of representations in earlier network levels can no longer be resolved by virtue of an object-unique binding node. Predictions of this model have been confirmed in several empirical studies and connectionist simulations (Bartko et al. 2007a,b, 2010; McTighe et al. 2010), and it readily explains why reducing sensory stimulation rescues amnesia in animals with lesions to perirhinal cortex (McTighe et al. 2010).

This theoretical framework could be extended to spatial representations, such that the hippocampus represents the top level of a hierarchical network in which representations from hierarchically lower layers are integrated into cognitive maps, and, in a much broader sense, contextual representations (O'Keefe and Nadel 1978; Eichenbaum 1999). For example, various kinds of spatially relevant information, such as distal cues, borders, geometry, visual scenes, and objects are represented in parahippocampal structures; that is, subiculum, entorhinal, postrhinal, and perirhinal cortex (Fyhn et al. 2004; Solstad et al. 2008; Deshmukh et al. 2012; Bett et al. 2013; Connor and Knierim 2017; Wang et al. 2018; Gofman et al. 2019; Høydal et al. 2019). These areas receive inputs from sensory areas, such that potential sources of interference for object location memories could arise from a large variety of sensory stimuli (Nadel and Hardt 2011). Our paradigm could be helpful in exploring these dynamics.

Like our findings, research in humans has shown that reducing sensory stimulation and mental activity after learning improves retention of spatial knowledge. These studies required participants to navigate virtual mazes or virtual towns and then tested retention of spatial knowledge at later time points. Immediately after learning the routes, participants either rested quietly for 10 min or performed cognitive tasks. Invariably, those who rested performed better than those who did not in tests assessing their knowledge of where landmarks were located from various vantage points (Craig et al. 2015, 2016a,b). Interestingly, when tested immediately after learning a route through a virtual town, participants made larger angular errors when pointing at the location of landmarks than when tested after the 10 min retention interval; this gain in accuracy, however, was notably stronger when they rested during the retention interval than when they engaged in a cognitive task (Craig et al. 2019). The object location recognition protocol we used here leads to long-term memories that require the hippocampus for as long as they can be expressed (Hardt et al. 2010; Migues et al. 2014, 2016). Thus, it would be interesting for future studies to explore whether reducing sensory stimulation after spatial learning increases the precision of spatial knowledge by assessing, for example, the coherence or spatial information content of place fields in the hippocampus.

During the time in sensory isolation, we took great care that rats did not fall asleep. To this end, we continuously observed the animals and intervened immediately by means of gentle handling, an established procedure widely used to interrupt onset of sleep in rodents without inducing a meaningful stress reaction (Meerlo and Turek 2001; van der Borght et al. 2006; Hagewoud et al. 2010a,b; Vecsey et al. 2013; Tudor et al. 2016). In our protocol, the earliest that rats required this intervention was 30–45 min after being placed in the box. Thus, our findings seem to replicate outcomes of studies with humans. However, whether quiet wakefulness, rather than sleep, promotes retention of the location of the objects cannot be concluded with certainty at present. Indeed, it has been demonstrated that rats that were allowed to sleep during a 2-h period after exploring two identical objects in an open field showed long-term location memory when tested 1 wk later in an object location recognition test, unlike rats that were kept awake (Sawangjit et al. 2018). Our findings, however, point to the interesting possibility that a process common to certain sleep phases and quiet wakefulness may promote long-term memory formation for memories that involve the hippocampus.

To conclude, we here provide a simple yet effective experimental procedure in rats that might be useful detecting the neurobiological processes that might explain how reducing sensory stimulation after learning enhances memory. Apart from this, our outcomes bear methodological implications for experimental protocol design; in many cases, animals return directly to their home cage after training, which can compromise memory retention, as our results suggest. Therefore, the experience of animals after the experimental task should be taken into consideration when designing experiments as it can affect the outcomes and the interpretation of results. Our observations in rats confirm a rich set of human findings, pointing to the possibility that ongoing sensory experience from everyday events in the period after learning might compromise memory stabilization processes, or memory consolidation, which may depend on the hippocampus, leading to the phenomenon of retroactive interference. Future research may tell us whether and which hippocampal processes play a critical role in this phenomenon, and, why damage to the hippocampus causes dense anterograde amnesia.

## Materials and Methods

### Subjects

Twenty-eight adult male Lister Hooded rats were obtained from Charles River Laboratories at 300–350 g and kept on a 12-h light–dark cycle (lights on 7 a.m.), with training and testing always performed in the light phase of the cycle. Animals were 3–8 mo old when experiments started. To maintain good levels of spontaneous exploration, animals were kept on a feeding regime to maintain their weight at 90%–95% of their free-feeding body weight, receiving ∼25–30 g of laboratory chow daily after each testing. Rats had free access to water. Animals used in experiments 1 and 3 were housed in cages of four. Animals used in experiment 2 had previously undergone surgery for chronic implantation of tetrodes in the hippocampus for a recording study (data not reported here) and were housed singly. The tetrode surgery occurred at least 4 wk prior to the start of experiment 2. All procedures complied with the UK Animals (Scientific Procedures) Act (1986) and the European Communities Council Directive of November 24, 1986 (86/609/EEC). All animal experiments were carried out in compliance with protocols approved by the University of Edinburgh Animal Welfare and Ethical Review Board (AWERB), and under a UK Home Office Project Licence.

### Apparatus

#### Video recording

Behavior was recorded using an overhead camera, through Blackmagic video capture software (Blackmagic Media Express version 3.3.1.).

#### Open field arena

The open field arena measured 60 × 60 cm, with wooden white walls 60 cm high and a wooden white floor. A striped black and white cue card (30 × 20 cm) was fixed to the North wall, and a variety of 2D and 3D cues attached either to the top of the walls or just outside the testing box in clear view. Cues were arranged in an asymmetric fashion, with one wall always devoid of cues. The floor of the open field arena was covered with the same type of bedding used in the home cages of the rats. Before each trial this bedding was disturbed to ensure no scent trails remained, and feces was removed. The luminance on the floor of the testing box was measured on its floor using a light meter, and the lights of the room were dimmed such that the testing box was at 20 ± 1 lumen.

#### Objects

Objects were of similar height or width (approximately 10 × 10 cm), but of varying textures, colors and shapes. Objects were made of nonporous and easily cleanable material such as ceramic, glass and metal. None of the objects had faces or pictures of animals that could have elicited an innate preference or anxiety response. Objects were fixed to clear mason glass bases (7 × 9 cm), which could be secured on the floor of the testing box for stability during exploration. Before a trial, objects were cleaned thoroughly with alcohol disinfectant wipes to remove any residual scents.

#### Postsampling enclosures

Home cage condition: The standard colony Home Cage (61 × 44 × 26 cm) was half plastic, half metal bars (Techniplast 2000P-224). Located on a trolley in the laboratory adjacent to the testing room, animals in the Home Cage were in an environment rich with various sensory cues. The cage mates remained in the Home Cage during the postsampling period. The Home Cage condition therefore provided a variety of sensory and social stimuli in order to produce a condition of high interference. Black Box condition: The box was made of black plastic, measured (38 × 32 × 24 cm), and was covered with a black plastic lid such that when inside the box the rat was in the dark. The floor was covered with the same type of bedding also used in the Home Cage and the open field arena. Rats were always alone in the Black Box. The Black Box was in a separate room; that is, not where the open field arena or the Home Cage were placed. The Black Box condition aimed to replicate the restful wake condition used in human studies, where participants were by themselves in a dark room. Dark Box condition: The Dark Box aimed to refine the conditions of the Black Box, in that it allowed the experimenter to view whether the rat was about to fall asleep. To this end, we used a sound-attenuating chamber (56 × 53 × 51 cm) with red light illumination (30 ± 5 lumens), a fan running constantly for ventilation, and a recording camera. We placed a covered plastic cage to house the rat inside this chamber (when it was alone: 42 × 27 × 40 cm; when it was with a cage mate: 60 × 44 × 30 cm). Rats cannot perceive red light (Szél and Röhlich 1992), so while the experimenter could observe the rat via a live video stream, the rat itself experienced darkness as in the Black Box. Light Box condition: To provide a comparable condition that permited visual stimulation, we changed the illumination in the chamber from red to normal (daylight-type) lighting and kept everything else the same.

### Procedures for experiment 1

Experiment 1 used a between-subjects design. All animals (*N* = 16) were pseudorandomly assigned to a group, ensuring an even split of groups within each home cage. Each animal underwent the experiment once.

#### Prehabituation

Before experiments began, rats participated in 5 d of gentle handling. Every day, all rats from a cage were placed together in a large plastic bin (50 × 92 × 41 cm) containing various objects, such as metal whisks, ceramic egg cups, and wooden objects. The bin was covered to a depth of ∼5 cm with the same type of bedding used for the home cages. Rats spent 30 min per day in the bin together, during which an experimenter removed each rat regularly by gently lifting the animal out of the bin, placing it briefly (10–20 sec) onto the lap, and then returning the rat slowly into the bin. This served to familiarize the animals to the experimental situation in which they soon would find themselves being placed into and removed out of an open field containing objects. The objects used during Prehabituation were not used during the actual experiments.

#### Habituation

Animals were first habituated to the Black Box. This consisted of each animal being placed individually into a Black Box and left there undisturbed for 1 h each day, for three consecutive days. We then continued to habituate the rats to both the open field and the Black Box, over four consecutive days. We transported the rats in a covered bucket and placed them into the empty open field with their snout facing one corner. During the four trials each corner was used once as a starting position for each animal in a random order. After 5 min of free exploration, we transferred the rats to the black boxes and left them there undisturbed for 1 h before returning them to their home cage. During these habituation trials, animals could sleep in the Black Box, to avoid a sleep deprivation effect. These seven days of exposing rats to both the experimental setup (i.e., 5-min open field) and the Black Box ensured that both groups were equally pre-exposed to the Black Box such that this experience of regular quiet wakefulness after exposure to the open field could not account for potentially better object location learning and memory in the Black Box rats.

#### Sampling

Sampling was carried out 24 h after the last habituation session. Two copies of the same novel object were placed in north (NW and NE) or south (SW and SE) positions. These positions were determined in a fully counterbalanced manner. The animal was placed into the testing box with their snout facing one corner and allowed to explore the objects for 5 min.

#### Postsampling condition

After sampling animals were transferred into either the black box or back into their home cage. Animals in the black box were kept awake with the widely used gentle handling technique (Meerlo and Turek 2001; van der Borght et al. 2006; Vecsey et al. 2013). This involved gently picking up the animal ∼2 in above the ground when the animal started to fall asleep, and then slowly and gently placing them down again. Because the experimenter in this study could not directly observe the rats, we peeked into the Black Box every 5 min or whenever the rat stopped making the typical audible noises (e.g., digging, moving around, etc.), and intervened with gentle handling if necessary. The method has been used previously in sleep deprivation studies and has been found to cause minimal amounts of stress (Colavito et al. 2013). After 1 h in the Black Box animals were returned to their home cage.

#### Probe

After a 6-h delay, which included either 6 h in the home cage or 1 h in the black box followed by 5 h in the home cage, rats participated in a Probe trial. For theProbe trial we moved one of the objects to a novel location, while one remained at the same place as in the sampling phase. The novel object location was positioned diagonally in relation to the familiar object location (i.e., NW and SE or NE and SW). We placed each rat into the same corner as in the sampling session and it was free to explore the open field for 3 min. We fully counterbalanced the positions of objects within and between groups.

### Procedures for experiment 2

Experiment 2 used a within-subjects design. All animals (*N* = 12) underwent the entire experiment twice; that is, they experienced the Dark Box and Light Box condition twice each. We used the two sequences (Light–dark)–(Dark–Light) and (Dark–Light)–(Light–dark). The first repetition of the two conditions was in a different context than the second one. In each repetition, we used the same object locations during sampling for the two box conditions, but two different objects and different novel locations in the probe sessions. We changed the objects as well as object locations during the sampling and probe sessions between repeats, such that sampling and novel object locations were not the same for the first and second repetition, and neither were the objects. This protocol ensured that novel object locations were different within and between repetitions. For each repetition, we allowed for at least 3 d between the end of one condition and the beginning of the next to decrease the possibility that rats would remember the object locations used in the previous repeats. The two repetitions were no less than 2 wk apart. We based this timeline on our previous studies documenting the forgetting curve for object locations in this task (Migues et al. 2016). We assigned rats randomly to conditions, and counterbalanced all objects, their locations, and experimental conditions between animals.

#### Prehabituation

Prehabituation was carried out as described in experiment 1.

#### Habituation

Animals were first habituated to both the Dark Box and the Light Box for three consecutive days. To this end, we placed each rat each day into a Dark Box and left it in there undisturbed for 1 h, followed by 1 h in a Light Box. We randomized the order in which animals were placed into these two boxes from day to day ensuring that animals in the end experienced both possible sequences. We then habituated the rats to the procedure they would encounter during Sampling. For four consecutive days, we placed rats into the open field for 10 min, and then placed them either into the Dark Box followed by the Light Box, or into the Light Box followed by the Dark Box, in a randomized order. During these habituation trials, we did not interfere with possible sleep occurring in the isolating boxes to avoid a possible sleep deprivation effect.

#### Sampling

As before, Sampling began 24 h after the last habituation session. We placed the rats into the open field arena for 10 min, where two copies of the same object were located in two adjacent corners (either at positions NW and NE, or SW and SE). We counterbalanced these positions within and between the experimental conditions.

#### Postsampling

After Sampling we transferred the rats either into the Dark Box or the Light Box for 1 h, depending on their experimental condition, which we assigned randomly. Rats were continuously monitored via a live video feed. In case a rat was about to fall asleep, we kept it awake using gentle handling as before. After 1 h in the box, we returned rats to their home cage.

#### Probe trial

After a 6-h delay, which included 1 h in either the dark or light box followed by 5 h in the home cage, rats were placed back into the testing box containing the two objects for 3 min, as outlined in experiment 1. The novel object location was positioned diagonally in relation to the familiar object location (i.e., NW and SE or NE and SW).

### Procedures for experiment 3

Experiment 3 used a within-subjects design. All animals (*N* = 8) underwent the entire experiment twice, with the test rats within each pair (see below) undergoing both conditions, in a pseudorandom order. Repeats of the experiment were no less than 1 wk apart to ensure that no memory for the previous object locations remained (Migues et al. 2016).

#### Prehabituation

Prehabituation was carried out as described in experiment 1.

#### Habituation

Rats were assigned into pairs for this experiment, with each home cage containing two pairs, and we randomly determined which rat would be tested and which rat would act as the social stimulus during sensory isolation after sampling. For three consecutive days, we placed both rats together into the Dark Box for 1 h, then removed the rat assigned as social stimulus rat and put it back into the home cage. The other rat remained in the Dark Box for another hour so it could get used to being in the Dark Box alone and being in the Dark Box with the cage mate. Then, for four consecutive days, we habituated the animals to the experimental procedure, putting the rat that would be tested alone into the open field for 10 min, and then into the Dark Box, either alone for 1 h and then with the cage mate for 1 h, or with the cage mate for 1 h and then alone for 1 h. We randomized the order in which animals were placed into these two conditions from day to day ensuring that animals in the end experienced both possible sequences. Rats could sleep during these habituation trials to avoid a sleep deprivation effect.

#### Sampling

As in the previous experiments, Sampling began 24 h after the last habituation session. We placed the test rat into the open field arena for 10 min with two copies of the same novel object located at two adjacent corners (either in position NW and NE, or SW and SE). We determined these positions randomly ensuring full counterbalancing.

#### Postsampling condition

Immediately after Sampling, we transferred the rats into the Dark Box for 1 h, either alone or together with their cage mate, depending on the assigned experimental condition, which we assigned randomly. As in the previous experiment, we monitored rats via a life video feed and kept them awake during this time via gentle handling in case they were about to fall asleep. After 1 h we returned the rats to their home cages.

#### Probe trial

Six hours after Sampling, we placed rats back into the open field arena, where we had moved one of the objects to a novel location, as described above. The novel object location was positioned diagonally in relation to the familiar object location (i.e., NW and SE or NE and SW).

### Analysis and statistics

We manually scored videos of the Sampling and Probe phases using our in-house software (zScore) to measure the time rats spent exploring the two objects. We considered a rat exploring an object when it was facing the object with its snout at least a 45° angle, while on the ground or on top of the object. If the rat was on top of the object, but not facing it (i.e., looking around the environment), we did not count it as object exploration. The experimenter was blind to both the postsampling condition of the rat and object novelty while scoring videos. We then calculated a discrimination index (*d*) that quantifies which object a rat preferred to explore: *d* = [(time exploring object at novel location) − (time exploring object at familiar location)]/(time exploring both objects). Values equal to 0 indicate that the rat has no preference, exploring both objects the same. Values >0 indicate that the rat prefers to explore the moved object. Because rats are naturally attracted to novelty, this index therefore allows to assess memory for the original object locations because only if the rat retained these it can determine what is novel in the open field. We required animals to explore objects for a minimum total of 20 sec to obtain a robust sample of their exploratory preferences. All animals reached this criterion both during Sampling and Probe, so none were excluded. We used GraphPad Prism (version 7.0, Graphpad) for data analysis. We examined whether data permitted parametric inferential tests using the Shapiro–Wilk normality test (experiment 1: Home Cage group, *W* = 0.91, *P* = 0.35, and Black Box group, *W* = 0.81, *P* = 0.03; experiment 2: Light Box group, *W* = 0.96, *P* = 0.81, and Dark Box group, *W* = 0.96, *P* = 0.82; experiment 3: Cage Mate group, *W* = 0.88, *P* = 0.17, and Alone group, *W* = 0.97, *P* = 0.91). Where data were normally distributed, we tested whether the discrimination indices were significantly different from zero using two-tailed one-sample *t*-tests. We used two-tailed unpaired *t*-tests for between group comparisons (experiment 1) and two-tailed paired *t*-tests for within group comparisons (experiments 2 and 3). We used *F* tests to compare the variances between groups or conditions (experiments 2 and 3). We used two-way ANOVAs to analyze overall differences in exploratory activity during Sampling and Probe between conditions. For data that were not normally distributed, we used nonparametric tests to determine significant effects, such as one-sample Wilcoxon rank test to determine whether the discrimination index was different from 0 and Welch's correction for *t*-tests to compare groups. We used our previously published results to estimate likely effect sizes and determine group sizes. We calculated both Cohen's *d* and partial η^2^ (η_p_^2^) to estimate the sizes of significant effects. Cohen's *d* allows to interpret the size of the difference between group means, based on the convention that *d* = 0.20 indicates small, *d* = 0.50 indicates medium, *d* = 80 indicates large, and *d* = 1.20 indicates very large effect sizes. η_p_^2^ provides a ratio of how much of the overall variance (i.e., effect plus error term) a particular variable explains, with η_p_^2^ = 0 denoting effects that collect no variance and η_p_^2^ = 1 denoting those that collect all of the variance in the dependent variable. We used the rank biserial correlation (*r*_*b*_) to determine the sizes of significant effects detected with the Wilcoxon rank test, conservatively assuming that *r*_b_ > 0.5 indicates large effects (Cohen 2013).
